# Assessment of nuclear grade-based recurrence risk classification in patients with hormone receptor-positive, human epidermal growth factor receptor 2-negative, node-positive high-risk early breast cancer

**DOI:** 10.1007/s12282-023-01500-2

**Published:** 2023-08-23

**Authors:** Takeshi Murata, Masayuki Yoshida, Sho Shiino, Chikashi Watase, Ayumi Ogawa, Shohei Shikata, Hiromi Hashiguchi, Yukiko Yoshii, Hirokazu Sugino, Kenjiro Jimbo, Akiko Maeshima, Eriko Iwamoto, Shin Takayama, Akihiko Suto

**Affiliations:** 1 Department of Breast Surgery, National Cancer Center Hospital, 5-1-1 Tsukiji, Chuo-ku, Tokyo, 104-0045 Japan; 2 Department of Diagnostic Pathology, National Cancer Center Hospital, 5-1-1 Tsukiji, Chuo-ku, Tokyo, 104-0045 Japan

**Keywords:** Breast cancer, Hormone receptor-positive, HER2-negative, Node-positive, High-risk of recurrence, Nuclear grade

## Abstract

**Background:**

Histological grade (HG) has been used in the MonrachE trial to select patients with hormone receptor (HR)-positive, human epidermal growth factor receptor 2 (HER2)-negative, node-positive high-risk early breast cancer (EBC). Although nuclear grade (NG) is widely used in Japan, it is still unclear whether replacing HG with NG can appropriately select high-risk patients.

**Methods:**

We retrospectively reviewed 647 patients with HR-positive, HER2-negative, node-positive EBC and classified them into the following four groups: group 1: ≥ 4 positive axillary lymph nodes (pALNs) or 1–3 pALNs and either grade 3 of both grading systems or tumors ≥ 5 cm; group 2: 1–3 pALNs, grade < 3, tumor < 5 cm, and Ki-67 ≥ 20%; group 3: 1–3 pALNs, grade < 3, tumor < 5 cm, and Ki-67 < 20%; and group 4: group 2 or 3 by HG classification but group 1 by NG classification. We compared invasive disease-free survival (IDFS) and distant relapse-free survival (DRFS) among the four groups using the Kaplan–Meier method with the log-rank test.

**Results:**

Group 1 had a significantly worse 5-year IDFS and DRFS than groups 2 and 3 (IDFS 80.8% vs. 89.5%, *P* = 0.0319, 80.8% vs. 95.5%, *P* = 0.002; DRFS 85.2% vs. 95.3%, *P* = 0.0025, 85.2% vs. 98.4%, *P* < 0.001, respectively). Group 4 also had a significantly worse 5-year IDFS (78.0%) and DRFS (83.6%) than groups 2 and 3.

**Conclusions:**

NG was useful for stratifying the risk of recurrence in patients with HR-positive, HER2-negative, node-positive EBC and was the appropriate risk assessment for patient groups not considered high-risk by HG classification.

**Supplementary Information:**

The online version contains supplementary material available at 10.1007/s12282-023-01500-2.

## Introduction

Hormone receptor (HR)-positive, human epidermal growth factor receptor 2 (HER2)-negative breast cancer is the most common subtype of all breast cancers that accounts for approximately 70% [1, 2]. Recently, the efficacy of cyclin-dependent kinase 4/6 (CDK 4/6) inhibitors as adjuvant therapy was demonstrated in patients with HR-positive, HER2-negative, node-positive high-risk early breast cancer (EBC) in the monarchE trial [3]. In the monarchE trial, the criteria for high risk were as follows: patients with ≥ 4 positive axillary lymph nodes (ALNs) or 1–3 positive ALNs and histological grade (HG) 3 or tumor ≥ 5 cm [3]. As indicated by this high-risk criterion, HG is one of the strong prognostic factors for patients with breast cancer [4]. The Nottingham combined HG, which consists of three components: tubule formation (TF) score, nuclear atypia (NA) score, and mitotic counts (MC) score, is currently the most commonly used grading system internationally. In this system, total scores of 3–5, 6 or 7, and 8 or 9 correspond to HG1, HG2, and HG3, respectively.

In Japan, most pathologists have incorporated the nuclear grade (NG), which is the sum of the NA and MC scores, alongside the HG in their assessment [5]. This grading system is a modification of the Black grading system [6] and was initially developed to distinguish patients at high risk of recurrence within the cohort of node-negative breast cancer patients [5]. In the NG system, total scores of 2 or 3, 4, and 5 or 6 correspond to NG1, NG2, and NG3, respectively [5]. Consequently, following the criteria of both grading paradigms, a patient with HG3 would have either have a score of 8 (TF score 2, NA score 3, and MC score 3) or 9 (TF score 3, NA score 3, and MC score 3), and still be classified as NG3 (NA score 3, and MC score 3, total score = 6).

The Japanese Breast Cancer Society Clinical Practice Guideline for the pathological diagnosis of breast cancer recommended “histological/nuclear” grading system in daily clinical practice [7]. Several studies have reported that HG3 and NG3 are significantly associated with a worse prognosis than the other grades [5, 8–10]. Particularly, in patients with ER-positive, HER2-negative breast cancer, both HG3 and NG3 had significantly worse outcomes [11–13]. Another report showed that the clinical outcomes of patients with NG3 tumors have proven to be significantly or near significantly worse than those of patients with NG2 tumors [14]. Among patients with HG2, there are cases with high TF score and low NA and MC scores (e.g., TF score 3, NA score 2, and MC score 1, total score = 6), and cases with low TF score and high NA and MC scores (e.g., TF score 1, NA score 3, and MC score 3, total score = 7). Therefore, they both receive different NG scores, with the former being classified as NG1 (NA score 2, MC score 1, total score = 3) and the latter as NG3 (NA score 3, MC score 3, total score = 6). Thus, by using the NG instead of the HG for grading, we can see that some patients with HG2 are classified as NG3 (i.e., patients with HG2/NG3). In fact, a report directly comparing the HG and NG systems showed that the overall concordance rate was more than 70%, unveiling instances where some patients with HG2 were classified as NG3, while none with NG2 were classified as HG3 [11].

Given the high concordance between the grading systems and the usefulness of HG3 and NG3 as prognostic factors, replacing the HG with the NG in the selection criteria for patients at a high risk of recurrence used in the monarchE trial may provide adequate risk stratification. However, such a shift might alter risk classification for patients with HG2/NG3. For example, a patient with HG2/NG3, two involved nodes, and a tumor size of 2 cm would not be classified as high-risk according to the HG but would be classified as high-risk according to the NG. However, these aspects have not yet been fully evaluated. Therefore, this study aimed to evaluate whether risk stratification by HG used in the monarchE study could also be achieved using risk stratification by NG. Furthermore, the study also aimed to focus on the prognosis of the patients whose risk cohort was altered by the NG system.

## Patients and methods

Overall, 647 HR-positive, HER2-negative, node-positive patients who were diagnosed with primary breast cancer at the National Cancer Center Hospital between January 2011 and December 2019 were identified. The exclusion criteria were as follows: (1) ductal carcinoma in situ at primary breast cancer, (2) stage IV disease at initial diagnosis, (3) patients without ALN involvement in primary breast cancer, and (4) those with unknown receptor status, NG, HG, and Ki-67 index. The medical records of the included patients were obtained from our prospectively generated database to extract the patient age at initial diagnosis, sex, clinical and pathological tumor size, clinical and pathological nodal status, HG, NG, histological type, menopausal status, estrogen receptor (ER) status, progesterone receptor (PR) status, HER2 status, Ki-67 index, presence or absence of lymphovascular invasion, type of initial surgery, chemotherapy, postoperative radiotherapy, and endocrine therapy (ET). For the evaluation of HG and NG, patients who underwent neoadjuvant chemotherapy (NACT) were evaluated using needle biopsy specimens before NACT, and those who did not undergo NACT were evaluated using surgical specimens. First, we defined three risk cohorts based on the risk classification of the monarchE study [3]. They were cohort 1: patients with ≥ 4 positive ALNs or 1–3 positive ALNs and grade 3 or tumors ≥ 5 cm; cohort 2: patients with 1–3 positive ALNs, grade < 3, tumor size < 5 cm, and high Ki-67 index (≥ 20%); and cohort 3: patients with 1–3 positive ALNs, grade < 3, tumor size < 5 cm, and low Ki-67 index (< 20%). Following this, all eligible patients were divided into four groups according to cohort conversion pattern by both grading systems; group 1: patients in cohort 1 by HG to cohort 1 by NG (i.e., no cohort conversion), group 2: patients in cohort 2 by HG to cohort 2 by NG (i.e., no cohort conversion), group 3: patients in cohort 3 by HG to cohort 3 by NG (i.e., no cohort conversion), and group 4: patients in cohort 2 or 3 by HG to cohort 1 by NG (i.e., cohort conversion). HR positivity was defined as either ER- or PR-positive. ER and PR were considered positive if the immunohistochemistry (IHC) staining was positive in > 1% of tumor cells [15]. A HER2 negative result corresponded to a score of 0 or 1+ on IHC or 2+ on IHC without amplification on fluorescence in situ hybridization [16–18]. The tumor, node, and metastasis staging of breast cancer was based on the 8th edition of the American Joint Committee on Cancer staging manual [19]. The Ki-67 index was evaluated using the same method in the monarchE study. Specifically, the Ki-67 index was measured in all untreated breast primary tumor samples using the Ki-67 IHC assay developed by Agilent Technologies (formerly Dako; Santa Clara, CA, USA) [3].

### Statistical analyses

Invasive disease-free survival (IDFS) and distant relapse-free survival (DRFS) among the four groups were estimated using the Kaplan–Meier method, and survival estimates were compared using the log-rank test. In addition, we also tried to evaluate IDFS and DRFS separately for patients who received NACT and those who did not. This differentiation was important as we took into account that HG and NG had been assessed in distinct specimens for these two patient groups. IDFS was defined as the time from the initial surgery date to the date of the first occurrence of ipsilateral invasive breast tumor recurrence, local/regional invasive breast cancer recurrence, distant recurrence, all-cause mortality, contralateral invasive breast cancer, or second primary non-breast neoplasm. DRFS was defined as the time from the initial surgery date to the date of distant recurrence or all-cause mortality, whichever occurred first. Cox proportional-hazards model with hazard ratios (HRs) and 95% confidence intervals (CIs) was used to evaluate the independent prognostic effects of each variable on IDFS and DRFS. The baseline variables (*P* < 0.05) in the univariate analysis were included in the multivariate analysis. Baseline characteristics were evaluated using the Mann–Whitney U test or chi-square test, as appropriate. All statistical analyses were conducted using the statistical software STATA SE version 16 (StataCorp LP, College Station, TX, USA), and statistical significance was set at *P* < 0.05.

### Ethical approval

The National Cancer Center Hospital Review Board and Ethical Committee approved this study (approval no. 2017-278), and the requirement for informed consent was waived because of the retrospective nature of the study.

## Results

### Patient demographics and tumor characteristics

**Fig. 1:**
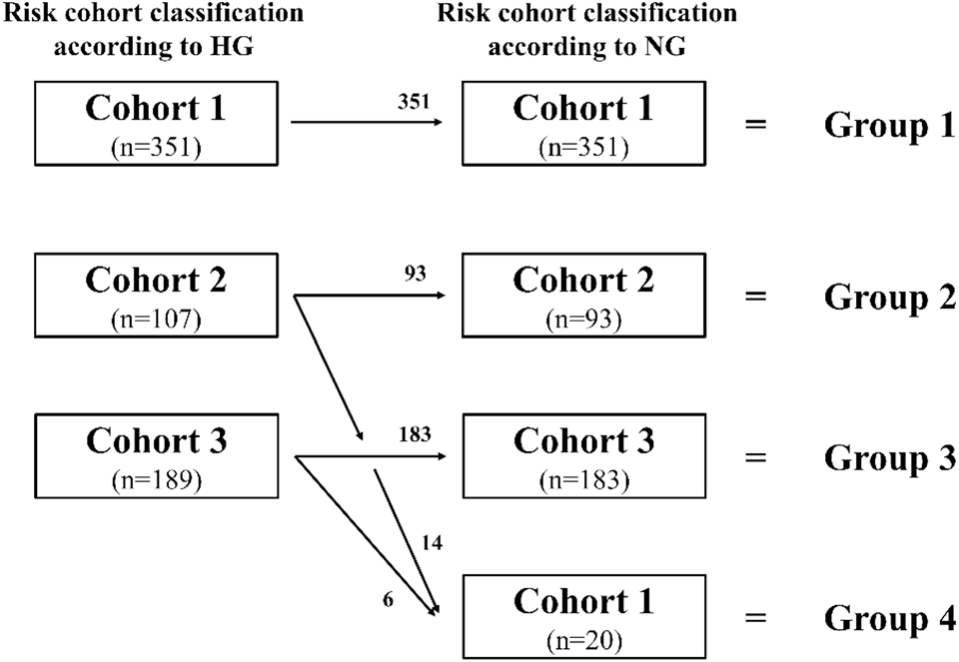
The relationship between the risk cohorts based on each grading system and the defined groups. Group 1: Patients in cohort 1 by HG to cohort 1 by NG (i.e., no cohort conversion). Group 2: Patients in cohort 2 by HG to cohort 2 by NG (i.e., no cohort conversion). Group 3: Patients in cohort 3 by HG to cohort 3 by NG (i.e., no cohort conversion). Group 4: Patients in cohort 2 or 3 by HG to cohort 1 by NG (i.e., cohort conversion). Cohort 1: Patients with ≥ 4 positive ALNs or 1–3 positive ALNs and grade 3 or tumors ≥ 5 cm. Cohort 2: Patients with 1–3 positive ALNs, grade < 3, tumor size < 5 cm, and high Ki-67 index (≥ 20%). Cohort 3: Patients with 1–3 positive ALNs, grade < 3, tumor size < 5 cm, and low Ki-67 index (< 20%). *HG* histological grade, *NG* nuclear grade, *ALNs* axillary lymph nodes

According to risk cohort classification by HG, 351 (54.3%), 107 (16.5%), and 189 (29.2%) patients were classified as cohorts 1, 2, and 3, respectively, while according to risk cohort classification by NG, 371 (57.3%), 93 (14.4%), and 183 (28.3%) patients were classified as cohorts 1, 2, and 3, respectively. Among the 647 patients, 351 (54.3%), 93 (14.4%), 183 (28.3%), and 20 (3.1%) patients were classified as groups 1, 2, 3, and 4, respectively. The relationship between the risk cohorts based on each grading system and the defined groups is shown in Fig. 1.The overall concordance rate between HG and NG was 70.3% (455/647). Particularly, the 193 patients with HG3 were classified as NG3, and 103 (92.0%) of the 112 patients with HG1 were classified as NG1. In contrast, 31 (9.1%) of the 342 patients with HG2 were classified as NG3 (Table 1). Of these 31 patients, 20 were classified as group 4 for NG3 (Fig. 1). Table 2 presents the demographics and tumor characteristics of all patients and each of the four groups. No differences were found in age, sex, ER status, HER2 status, or ET rates among the four groups. The PR-negative status was significantly higher in group 1 than in group 3, and the total mastectomy rates were significantly higher in group 1 than in groups 2 and 3. Additionally, the axillary lymph node dissection (ALND) rate was significantly higher in group 1 than in groups 3 and 4. However, chemotherapy and irradiation were significantly more common in group 1 than in groups 2, 3, and 4.

**Table 1:** Histological grades and nuclear grades of all patients

	HG1	HG2	HG3	Total
NG1	103	152	0	255 (39.4%)
NG2	9	159	0	168 (26.0%)
NG3	0	31	193	224 (34.6%)
Total	112 (17.3%)	342 (52.9%)	193 (29.8%)	647

*NG* nuclear grade, *HG* histological grade

**Table 2:** Patients’ characteristics

Category	All patients (n = 647)		Group 1 (n = 351)	Group 2 (n = 93)	Group 3 (n = 183)	Group 4 (n = 20)	*p* value Group 1 vs Group 2	*p* value Group 1 vs Group 3	*p* value Group 1 vs Group 4
	n (%)		n (%)	n (%)	n (%)	n (%)			
Age, years									
< 65	500 (77.3)		265 (75.5)	79 (85.0)	140 (77.3)	16 (80.0)	0.052	0.797	0.648
≥ 65	147 (22.7)		86 (24.5)	14 (15.1)	43 (23.5)	4 (20.0)			
Sex									
Female	642 (99.2)		348 (99.1)	91 (97.8)	183 (100)	20 (100)	0.292	0.210	0.678
Male	5 (0.8)		3 (0.9)	2 (2.2)	0 (0)	0 (0)			
Menopausal status									
Pre	321 (49.6)		165 (47.0)	51 (54.8)	91 (49.7)	14 (70.0)	0.179	0.551	0.045
Post	326 (50.4)		186 (53.0)	42 (45.2)	92 (50.3)	6 (30.0)			
ER status									
Positive	642 (99.2)		346 (98.6)	93 (100)	183 (100)	20 (100)	0.247	0.105	0.591
Negative	5 (0.8)		5 (1.4)	0 (0)	0 (0)	0 (0)			
PR status									
Positive	590 (91.2)		309 (88.0)	86 (92.5)	176 (96.2)	19 (95.0)	0.224	0.002	0.344
Negative	57 (8.8)		42 (12.0)	7 (7.5)	7 (3.8)	1 (5.0)			
HER2 status									
0	228 (35.2)		124 (35.3)	31 (33.3)	70 (38.3)	3 (15.0)	0.720	0.505	0.062
1+ , 2+ /ISH-	419 (64.8)		227 (64.7)	62 (66.7)	113 (61.7)	17 (85.0)			
HG									
Grade 1	112 (17.3)		28 (8.0)	11 (11.8)	73 (39.9)	0 (0)	< 0.001	< 0.001	< 0.001
Grade 2	342 (52.9)		130 (37.0)	82 (88.2)	110 (60.1)	20 (100)			
Grade 3	193 (29.8)		193 (55.0)	0 (0)	0 (0)	0 (0)			
NG									
Grade 1	255 (39.4)		85 (24.2)	34 (36.6)	136 (74.3)	0 (0)	< 0.001	< 0.001	0.001
Grade 2	168 (26.0)		62 (17.7)	59 (63.4)	47 (25.7)	0 (0)			
Grade 3	224 (34.6)		204 (58.1)	0 (0)	0 (0)	20 (100)			
Ki-67 index, %									
< 20	295 (45.6)		106 (30.2)	0 (0)	183 (100)	6 (30.0)	< 0.001	< 0.001	0.985
≥ 20	352 (54.4)		245 (69.8)	93 (100)	0 (0)	14 (70.0)			
Number of positive nodes									
1–3	463 (71.6)		167 (47.6)	93 (100)	183 (100)	20 (100)	< 0.001	< 0.001	< 0.001
≥ 4	184 (28.4)		184 (52.4)	0 (0)	0 (0)	0 (0)			
Tumor size, cm									
< 2	241 (37.3)		86 (24.5)	42 (45.2)	103 (56.3)	10 (50.0)	< 0.001	< 0.001	0.005
2–5	305 (47.1)		164 (46.7)	51 (54.8)	80 (43.7)	10 (50.0)			
≥ 5	101	(15.6)	101 (28.8)	0 (0)	0 (0)	0 (0)			
Breast surgery									
BCS	219 (33.9)		94 (26.8)	38 (40.9)	81 (44.3)	6 (30.0)	0.008	< 0.001	0.752
TM	428 (66.2)		257 (73.2)	55 (59.1)	102 (55.7)	14 (70.0)			
Axillary surgery									
SLNB only	65 (10.1)		16 (4.6)	7 (7.5)	38 (20.8)	4 (20.0)	0.251	< 0.001	0.003
ALND	582 (90.0)		335 (95.4)	86 (92.5)	145 (79.2)	16 (80.0)			
CT									
No	179 (27.7)		46 (13.1)	17 (18.3)	107 (58.5)	9 (45.0)	0.022	< 0.001	< 0.001
Neoadjuvant	84 (13.0)		72 (20.5)	8 (8.6)	2 (1.1)	2 (5.0)			
Adjuvant	384 (59.4)		233 (66.4)	68 (73.1)	74 (40.4)	9 (45.0)			
RT									
No	238 (36.8)		73 (20.8)	49 (52.7)	103 (56.3)	13 (65.0)	< 0.001	< 0.001	< 0.001
Yes	409 (63.2)		278 (79.2)	44 (47.3)	80 (43.7)	7 (35.0)			
ET									
No	25 (3.9)		13 (3.7)	2 (2.2)	9 (4.9)	1 (5.0)	0.461	0.503	0.767
Yes	622 (96.1)		338 (96.3)	91 (97.8)	174 (95.1)	19 (95.0)			

Group 1: Patients in cohort 1 by HG to cohort 1 by NG (i.e., no cohort conversion)

Group 2: Patients in cohort 2 by HG to cohort 2 by NG (i.e., no cohort conversion)

Group 3: Patients in cohort 3 by HG to cohort 3 by NG (i.e., no cohort conversion)

Group 4: Patients in cohort 2 or 3 by HG to cohort 1 by NG (i.e., cohort conversion)

Cohort 1: Patients with ≥ 4 positive ALNs or 1–3 positive ALNs and grade 3 or tumors ≥ 5 cm

Cohort 2: Patients with 1–3 positive ALNs, grade < 3, tumor size < 5 cm, and high Ki-67 index (≥ 20%)

Cohort 3: Patients with 1–3 positive ALNs, grade < 3, tumor size < 5 cm, and low Ki-67 index (< 20%)

*HR* hazard ratio, *IDFS* invasive disease-free survival, *DRFS* distant relapse-free survival, *CI* confidence interval, *ER* estrogen receptor, *PR* progesterone receptor, *HER2* human epidermal growth factor receptor 2, *CT* chemotherapy, *RT* radiotherapy, *ET* endocrine therapy, *BCS* breast-conserving surgery, *TM* total mastectomy, *SLNB* sentinel lymph node biopsy, *ALNs* axillary lymph nodes, *ALND* axillary lymph node dissection, *NG* nuclear grade, *HG* histological grade, *ISH* in situ hybridization

### Recurrence events

During the median follow-up of 71.4 months (interquartile range 51.4–98.5 months), 111 IDFS and 79 DRFS events occurred in all patients (Table 3). Of the 351 patients in group 1, most IDFS events were distant recurrences, and common sites of distant recurrence were the bone, liver, lung, and distant lymph nodes. Among the patients in group 2, most IDFS events were locoregional recurrence, distant recurrence, and second primary neoplasm, whereas, among those in group 3, most IDFS events were second primary neoplasms. Among the 20 patients in group 4, most IDFS events were distant recurrences. However, a small number of DRFS events occurred in groups 2, 3, and 4.

**Table 3:** Recurrence events

	All patients (n = 647)	Group 1 (n = 351)	Group 2 (n = 93)	Group 3 (n = 183)	Group 4 (n = 20)
IDFS events					
Total IDFS events	111	78	12	16	5
Patients with invasive disease, first occurrence	108	76	12	15	5
Local/regional recurrence	26	17	4	4	1
Distant recurrence	67	57	4	3	3
Contralateral recurrence	4	2	0	2	0
Second primary neoplasm	20	8	4	7	1
All-cause mortality without invasive disease	3	2	0	1	0
DRFS events					
Total DRFS events	79	63	5	7	4
Patients with distant relapse, any time	69	59	4	3	3
Bone	40	34	1	3	2
Liver	16	14	0	1	1
Lung	15	12	2	1	0
Brain	3	3	0	0	0
Lymph node	17	16	0	1	0
Pleura	1	1	0	0	0
CNS	2	1	1	0	0
Other^a^	2	2	0	0	0
All-cause mortality without distant recurrence	10	4	1	4	1

Some patients were counted more than once in the subcategories if they had recurrences at different locations

Group 1: Patients in cohort 1 by HG to cohort 1 by NG (i.e., no cohort conversion)

Group 2: Patients in cohort 2 by HG to cohort 2 by NG (i.e., no cohort conversion)

Group 3: Patients in cohort 3 by HG to cohort 3 by NG (i.e., no cohort conversion)

Group 4: Patients in cohort 2 or 3 by HG to cohort 1 by NG (i.e., cohort conversion)

Cohort 1: Patients with ≥ 4 positive ALNs or 1–3 positive ALNs and grade 3 or tumors ≥ 5 cm

Cohort 2: Patients with 1–3 positive ALNs, grade < 3, tumor size < 5 cm, and high Ki-67 index (≥ 20%)

Cohort 3: Patients with 1–3 positive ALNs, grade < 3, tumor size < 5 cm, and low Ki-67 index (< 20%)

*IDFS* invasive disease-free survival, *DRFS* distant relapse-free survival, *CNS* central nerve system, *IQR* interquartile range

^a^Includes stomach (two), Median follow-up 71.4 months (IQR 51.4–98.5)

### IDFS and DRFS based on the four groups

**Fig. 2:**
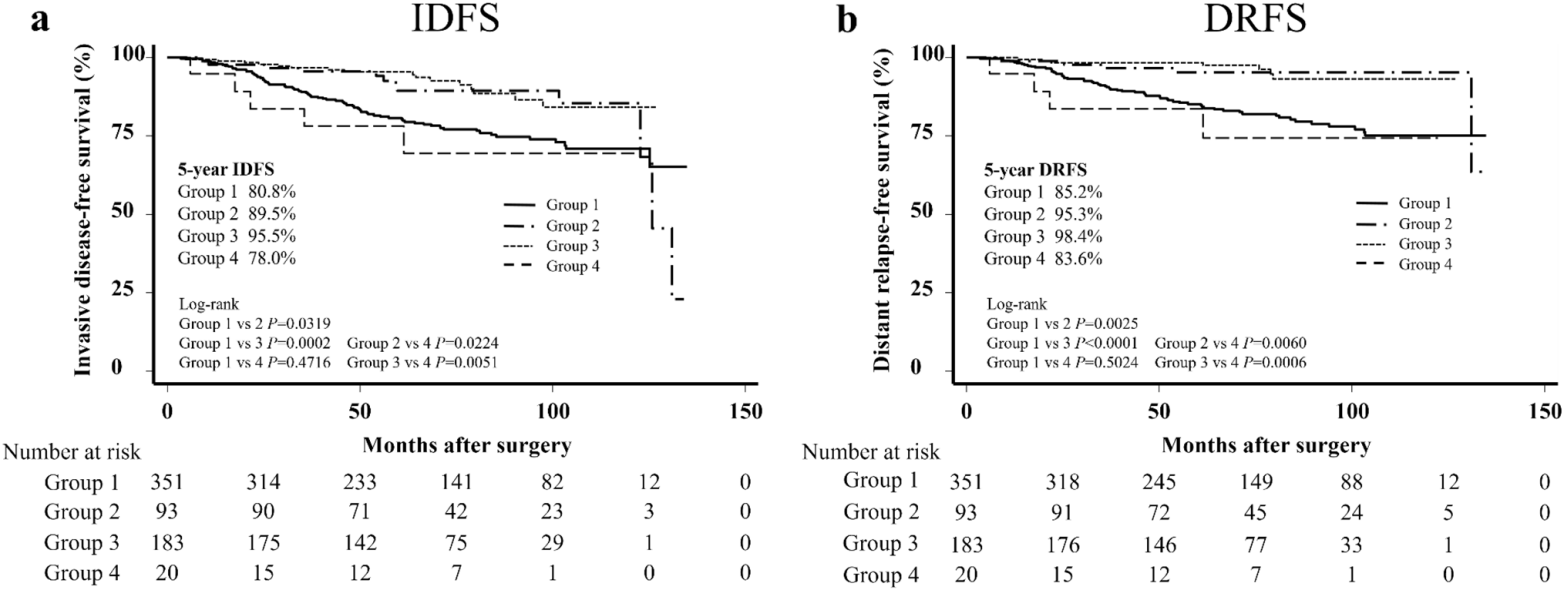
**a** Invasive disease-free survival (IDFS) and **b** distant relapse-free survival (DRFS) according to the risk group. Group 1: Patients in cohort 1 by HG to cohort 1 by NG (i.e., no cohort conversion). Group 2: Patients in cohort 2 by HG to cohort 2 by NG (i.e., no cohort conversion). Group 3: Patients in cohort 3 by HG to cohort 3 by NG (i.e., no cohort conversion). Group 4: Patients in cohort 2 or 3 by HG to cohort 1 by NG (i.e., cohort conversion). Cohort 1: Patients with ≥ 4 positive ALNs or 1–3 positive ALNs and grade 3 or tumors ≥ 5 cm. Cohort 2: Patients with 1–3 positive ALNs, grade < 3, tumor size < 5 cm, and high Ki-67 index (≥ 20%). Cohort 3: Patients with 1–3 positive ALNs, grade < 3, tumor size < 5 cm, and low Ki-67 index (< 20%). *IDFS* invasive disease-free survival, *DRFS* distant relapse-free survival, *GS* grading system, *HG* histological grade, *NG* nuclear grade, *ALNs* axillary lymph nodes, *CI* confidence interval

Regarding IDFS, patients in group 1 had significantly worse 5-year IDFS than those in groups 2 and 3 (80.8% vs. 89.5%, *P* = 0.0319; 80.8% vs. 95.5%, *P* = 0.0002, respectively). Patients in group 4 also had significantly worse 5-year IDFS than those in groups 2 and 3 (78.0% vs. 89.5%, *P* = 0.0224; 78.0% vs. 95.5%, *P* = 0.0051, respectively) (Fig. 2a). Univariate analysis revealed that the significant risk factors associated with IDFS events were patients with group 1 (HR 2.87; 95% CI 1.63–5.08; *P* < 0.001), group 4 (HR 3.25; 95% CI 1.05–10.0; *P* = 0.040), PR-negative status (HR 1.94; 95% CI 1.08–3.47; *P* = 0.027), and no ET (HR 2.73; 95% CI 1.42–5.24; *P* = 0.003). In the multivariate analysis, the significant risk factors for IDFS events were patients with group 1 (HR 2.84; 95% CI 1.64–4.92; *P* < 0.001) and no ET (HR 2.71; 95% CI 1.29–5.70; *P* = 0.009). Patients with group 4 were a borderline significant risk factor for IDFS (HR 3.08; 95% CI 0.97–9.81; *P* = 0.057) (Table 4a). Regarding DRFS, patients in group 1 had significantly worse 5-year DRFS than those in groups 2 and 3 (85.2% vs. 95.3%, *P* = 0.0025; 85.2% vs. 98.4%, *P* < 0.0001, respectively). Patients in group 4 also had significantly worse 5-year IDFS than those in groups 2 and 3 (83.6% vs. 95.3%, *P* = 0.0060; 83.6% vs. 98.4%, *P* = 0.0006, respectively) (Fig. 2b). Univariate analysis revealed that the significant factors associated with DRFS events were patients with group 1 (HR 10.7; 95% CI 3.37–34.1; *P* < 0.001), group 4 (HR 11.4; 95% CI 2.28–56.7; *P* = 0.003), PR-negative status (HR 2.31; 95% CI 1.20–4.44; *P* = 0.012), no radiotherapy (HR 0.46; 95% CI 0.26–0.81, *P* = 0.008), and no ET (HR 2.60; 95% CI 1.13–5.92; *P* = 0.024). In the multivariate analysis, the significant risk factors for DRFS events were patients with group 1 (HR 9.70; 95% CI 3.23–29.1; *P* < 0.001), group 4 (HR 11.0; 95% CI 2.15–56.4; *P* = 0.004), and no ET (HR 2.73; 95% CI 1.08–6.93; *P* = 0.034) (Table 4b).

**Table 4:** Univariate and multivariate analysis results of factors associated with invasive disease-free survival (IDFS) (a) and distant relapse-free survival (DRFS) (b)

Factor	Category	Univariate		Multivariate	
		HR (95% CI)	*p* value	HR (95% CI)	*p* value
(a) IDFS					
Risk Group	Group 3	Reference		Reference	
	Group 1	2.87 (1.63–5.08)	< 0.001	2.84 (1.64–4.92)	< 0.001
	Group 2	1.42 (0.67–3.01)	0.363	1.43 (0.70–3.02)	0.345
	Group 4	3.25 (1.05–10.0)	0.040	3.08 (0.97–9.81)	0.057
Age, years	< 65	Reference			
	≥ 65	1.25 (0.79–1.99)	0.335		
Menopausal status	Pre	Reference			
	Post	1.42 (0.96–2.09)	0.076		
ER status	Positive	Reference			
	Negative	1.37 (0.17–11.0)	0.768		
PR status	Positive	Reference		Reference	
	Negative	1.94 (1.08–3.47)	0.027	1.58 (0.87–2.85)	0.131
HER2 status	0	Reference			
	1+ , 2+ /ISH-	0.71 (0.48–1.05)	0.087		
Breast surgery	BCS	Reference			
	TM	1.46 (0.95–2.26)	0.085		
Axillary surgery	SLNB only	Reference			
	ALND	0.91 (0.57–1.45)	0.392		
CT	Yes	Reference			
	No	1.10 (0.69–1.75)	0.688		
RT	Yes	Reference			
	No	0.72 (0.57–1.45)	0.120		
ET	Yes	Reference		Reference	
	No	2.73 (1.42–5.24)	0.003	2.71 (1.29–5.70)	0.009
(b) DRFS					
Risk Group	Group 3	Reference		Reference	
	Group 1	10.7 (3.37–34.1)	< 0.001	9.59 (3.22–28.6)	< 0.001
	Group 2	2.45 (0.56–10.6)	0.233	2.46 (0.58–10.4)	0.222
	Group 4	11.4 (2.28–56.7)	0.003	11.0 (2.15–56.4)	0.004
Age, years	< 65	Reference			
	≥ 65	1.10 (0.62–1.96)	0.738		
Menopausal status	Pre	Reference			
	Post	1.31 (0.82–2.11)	0.263		
ER status	Positive	Reference			
	Negative	2.08 (0.27–16.3)	0.486		
PR status	Positive	Reference		Reference	
	Negative	2.31 (1.20–4.44)	0.012	1.67 (0.85–3.27)	0.133
HER2 status	0	Reference			
	1+ , 2+ /ISH-	0.72 (0.44–1.17)	0.182		
Breast surgery	BCS	Reference			
	TM	1.64 (0.95–2.83)	0.078		
Axillary surgery	SLNB only	Reference			
	ALND	6.34 (0.88–45.5)	0.066		
CT	Yes	Reference			
	No	0.52 (0.26–1.02)	0.058		
RT	Yes	Reference		Reference	
	No	0.46 (0.26–0.81)	0.008	0.76 (0.43–1.33)	0.335
ET	Yes	Reference		Reference	
	No	2.60 (1.13–5.92)	0.024	2.73 (1.08–6.93)	0.034

Group 1: Patients in cohort 1 by HG to cohort 1 by NG (i.e., no cohort conversion)

Group 2: Patients in cohort 2 by HG to cohort 2 by NG (i.e., no cohort conversion)

Group 3: Patients in cohort 3 by HG to cohort 3 by NG (i.e., no cohort conversion)

Group 4: Patients in cohort 2 or 3 by HG to cohort 1 by NG (i.e., cohort conversion)

Cohort 1: Patients with ≥ 4 positive ALNs or 1–3 positive ALNs and grade 3 or tumors ≥ 5 cm

Cohort 2: Patients with 1–3 positive ALNs, grade < 3, tumor size < 5 cm, and high Ki-67 index (≥ 20%)

Cohort 3: Patients with 1–3 positive ALNs, grade < 3, tumor size < 5 cm, and low Ki-67 index (< 20%)

*HR* hazard ratio, *IDFS* invasive disease-free survival, *DRFS* distant relapse-free survival, *CI* confidence interval, *ER* estrogen receptor, *PR* progesterone receptor, *HER2* human epidermal growth factor receptor 2, *CT* chemotherapy, *RT* radiotherapy, *ET* endocrine therapy, *BCS* breast-conserving surgery, *TM* total mastectomy, *SLNB* sentinel lymph node biopsy, *ALNs* axillary lymph nodes, *ALND* axillary lymph node dissection, *NG* nuclear grade, *HG* histological grade

This study included 84 and 563 patients who underwent and did not undergo NACT, respectively. The number of patients who received NACT was 72, 8, 2, and 2 for groups 1, 2, 3, and 4, respectively; IDFS events occurred in 24, 0, 0, and 1 patients, respectively, while DRFS events occurred in 20, 0, 0, and 1 patients, respectively (Online Resource Table 1). The number of events in groups other than group 1 was relatively too small to perform an adequate evaluation. In contrast, the numbers of patients who did not receive NACT were 279, 85, 181, and 18 for groups 1, 2, 3, and 4, respectively; IDFS events occurred in 54, 12, 16, and 4 patients, respectively, and DRFS occurred in 43, 5, 7, and 3 patients, respectively (Online Resource Table 2). The 5-year IDFS rates for groups 1 and 4 were significantly lower than that of group 3 (84.2% vs. 95.4%, *P* = 0.0067; 81.6% vs. 95.4%, *P* = 0.0282, respectively). Although the 5-year IDFS rate for group 2 was not significantly different from those for groups 1 and 4, there was a slightly significant trend (84.2% vs. 88.2%, *P* = 0.2728; 81.6% vs. 88.2%, *P* = 0.0953, respectively) (Online Resource Fig. 1a). Regarding DRFS, patients in group 1 had significantly worse 5-year DRFS than those in groups 2 and 3 (88.1% vs. 94.8%, *P* = 0.0269; 88.1 vs. 98.3%, *P* = 0.0004, respectively). Patients in group 4 also had significantly worse 5-year IDFS than those in groups 2 and 3 (87.8% vs. 94.8%, *P* = 0.0424; 87.8% vs. 98.3%, *P* = 0.0079, respectively) (Online Resource Fig. 1b).

## Discussion

In this study, we investigated whether NG, instead of HG, could appropriately stratify the prognosis of patients with HR-positive, HER2-negative, node-positive high-risk EBC, as defined in the monarchE trial. The results showed that the risk cohort classification by NG was highly consistent with that by HG, indicating that the risk classification by NG could appropriately stratify patients at a high risk of recurrence. Furthermore, patients classified as low-risk according to the HG classification but high-risk according to the NG classification (i.e., group 4) had a poor prognosis similar to those classified as high-risk according to both the HG and NG classifications (i.e., group 1). We could not fully evaluate the utility of NG in patients with NACT because of the small number of patients in this population (only 86 patients). However, among patients who did not undergo NACT, patients in group 4 also had poor prognoses similar to those in group 1, with respect to IDFS and DRFS. These results suggest that, at least for patients not undergoing NACT, NG can appropriately stratify prognosis even if it replaces HG and that it can also adequately select high-risk cases undetectable by HG. To the best of our knowledge, these results are the first to be reported.

The patients’ background in this study showed the following characteristics compared with those of the ET-alone group in the monarchE study [3]. Patients in the ET-alone group in the monarchE study were those in groups 1 and 2 in our study. However, compared with patients in the MonarchE study, our study patients had a higher proportion of those with 1–3 lymph nodes (59.8% vs. 40.4%), ≥ 65 years (22.7% vs. 14.6%), no chemotherapy (15.1% vs. 4.7%), and Ki-67 values of ≥ 20% (76.9% vs. 43.6%), respectively. In contrast, the clinicopathological factors, such as menopausal status, tumor diameter, HG, and ER/PR status, were similar between the monarchE study and this study. Regarding the follow-up period of this study, the median follow-up was > 70 months, which was sufficiently long to detect the occurrence of early recurrence events. The proportion of group 1 in the total patient population was > 50%, and we believe it a suitable target population to examine whether the stratification of recurrence risk could be replicated by replacing HG with NG. The overall concordance rate of HG and NG in this study was as high as 70.3% (455/647), particularly because all patients with HG3 were classified as those with NG3. This result is consistent with that of a previous report [11]. Although NG was used as a selection criterion for patients with a high risk of recurrence, the risk of underestimating the number of high-risk patients based on the monarchE study criteria was low. In contrast, 31 patients with HG2 were classified as those with NG3 in this study, and the risk cohort was changed to 20 of these patients (group 4). These patients in group 4 had a poor prognosis similar to those in group 1. Multivariate analyses showed that patients in group 4 were a significantly poor prognostic factor for DRFS and a marginally poor prognostic factor for IDFS.

Comparing the clinicopathological characteristics between the 31 and 311 patients with HG2/NG3 and HG2/NG1 or NG2, respectively, in this study showed that patients with HG2/NG3 had significantly lower tubule formation scores than those with HG2/NG1 or NG2, whereas the nuclear atypia score, mitotic count score, and Ki-67 value were significantly higher (Online Resource Table 3). Mitotic counts and Ki-67 values are commonly used to evaluate the proliferative activity of breast cancer, and tumor cell proliferative activity is an important independent prognostic factor in patients with breast cancer [20, 21]. Therefore, patients with HG2/NG3 have poorer prognostic factors than those with HG2/NG1 or NG2. This indicated a certain number of patients with NG3 who might be at a high risk of recurrence even if they were not determined to be at a high risk of recurrence because of HG2. Furthermore, the possibility that these patients have a poor prognosis suggests that the NG classification can identify a population that the conventional HG classification cannot adequately stratify. Although the evaluation of the three-grade classification scale may vary among pathologists, several studies have reported on the moderate reproducibility of HG with both inter- and intra-observer concordance [22–24]. Regarding NG, various activities have been conducted to standardize the criteria for NG assessment among pathologists, and the interobserver agreement level was also satisfactory [25–27].

This study had some limitations. First, this was a retrospective study performed at a single institution. Second, although we collected data from consecutive patients with HR-positive, HER2-negative, node-positive EBC, we did not adjust for a selection bias. Third, patients with unknown Ki-67 values were excluded from this study. Third, it is unclear whether CDK 4/6 inhibitors may benefit patients at a high risk of recurrence according to the NG classification. Finally, because of the relatively small sample size of this study, the reproducibility of the results needs to be validated with a larger sample size in a multicenter setting.

In conclusion, we showed that NG could be used to stratify the risk of recurrence of HR-positive, HER2-negative, node-positive EBC. Additionally, we demonstrated that NG could be used to select a group of patients who would not be considered high-risk if HG were used. Therefore, these results may contribute to adequate decision-making regarding adjuvant therapy according to the risk of recurrence.

### Supplementary Information

Below is the link to the electronic supplementary material.

Supplementary file1 (DOCX 289 KB)

Supplementary file2 (DOCX 19 KB)

Supplementary file3 (DOCX 20 KB)

Supplementary file4 (DOCX 21 KB)

## Data Availability

The datasets analyzed during the current study are available from the corresponding author on reasonable request.
