# Supplementary material for: Assessment of nuclear grade-based recurrence risk classification in patients with hormone receptor-positive, human epidermal growth factor receptor 2-negative, node-positive high-risk early breast cancer
Source: Breast Cancer. 2023 Aug 23;30(6):1054–64. doi: 10.1007/s12282-023-01500-2 (PMC10587205; doi:10.1007/s12282-023-01500-2)
Supplement: Supplementary file 1 — Supplementary file1 (DOCX 289 KB) [file 12282_2023_1500_MOESM1_ESM.docx]

*Breast Cancer*

**Assessment of nuclear grade-based recurrence risk classification in patients with hormone receptor-positive, human epidermal growth factor receptor 2-negative, node-positive high-risk early breast cancer**

Takeshi Murata^a*^, [tamurata@ncc.go.jp](mailto:tamurata@ncc.go.jp)

Masayuki Yoshida^b^, Sho Shiino^a^, Chikashi Watase^a^, Shohei Shikata^a^, Hiromi Hashiguchi^a^, Yukiko Yoshii^a^, Ayumi Ogawa^a^, Hirokazu Sugino^b^, Kenjiro Jimbo^a^, Akiko Maeshima^b^, Eriko Iwamoto^a^, Shin Takayama^a^, Akihiko Suto^a^

^a^Department of Breast Surgery, National Cancer Center Hospital, 5-1-1 Tsukiji, Chuo-ku, Tokyo 104-0045, Japan.

^b^Department of Diagnostic Pathology, National Cancer Center Hospital, 5-1-1 Tsukiji, Chuo-ku, Tokyo 104-0045, Japan.

***Corresponding author**

Takeshi Murata

Department of Breast Surgery, National Cancer Center Hospital, 5-1-1 Tsukiji, Chuo-ku, Tokyo 104-0045, Japan

Tel: +81-3-3547-5201

Fax: +81-3-3542-3815

E-mail: [tamurata@ncc.go.jp](mailto:tamurata@ncc.go.jp)

ORCID: 0000-0003-0942-7599

**Online Resource Fig. 1 (a) Invasive disease-free survival (IDFS) and (b) distant relapse-free survival (DRFS) according to risk group in patients who did not receive NACT**

**
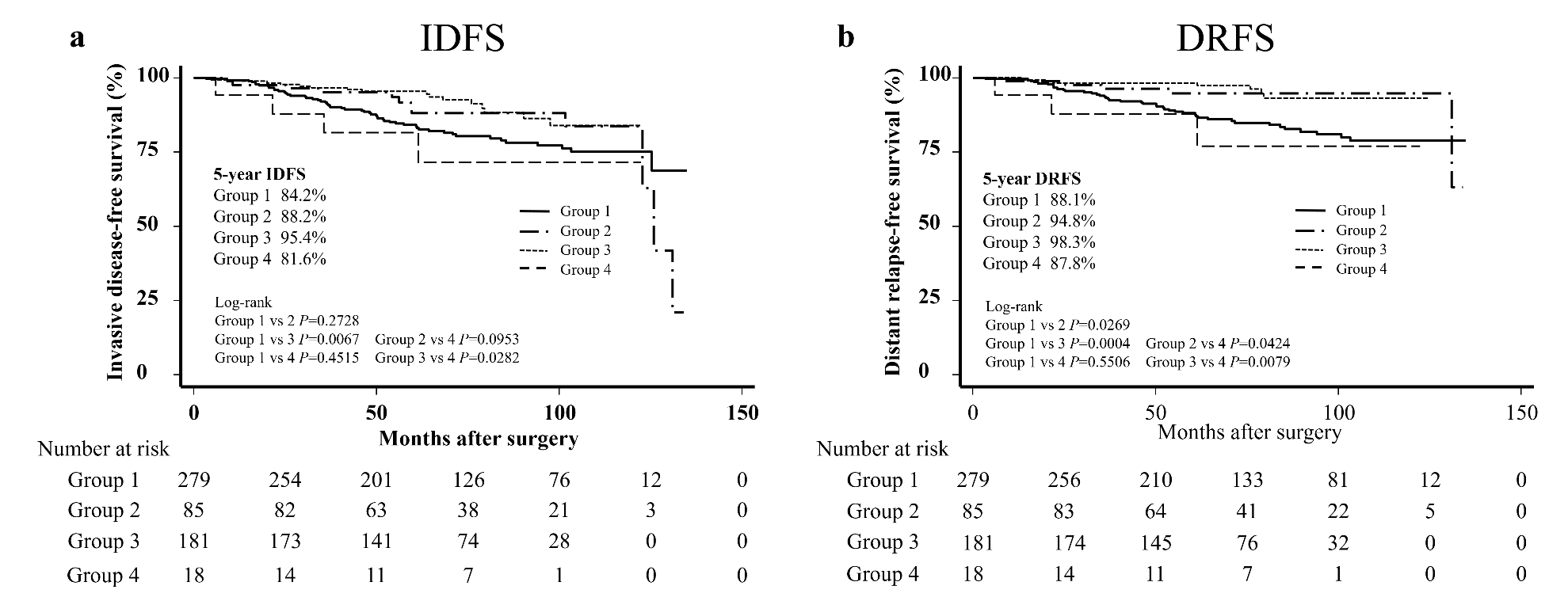
**

Group 1: Patients in cohort 1 by HG to cohort 1 by NG (i.e., no cohort conversion)

Group 2: Patients in cohort 2 by HG to cohort 2 by NG (i.e., no cohort conversion)

Group 3: Patients in cohort 3 by HG to cohort 3 by NG (i.e., no cohort conversion)

Group 4: Patients in cohort 2 or 3 by HG to cohort 1 by NG (i.e., cohort conversion).

Cohort 1: Patients with ≥4 positive ALNs or 1–3 positive ALNs and grade 3 or tumors ≥5 cm

Cohort 2: Patients with 1–3 positive ALNs, grade <3, tumor size <5 cm, and high Ki-67 index (≥20%)

Cohort 3: Patients with 1–3 positive ALNs, grade <3, tumor size <5 cm, and low Ki-67 index (<20%).

Abbreviations: IDFS, invasive disease-free survival; DRFS, distant relapse-free survival; GS, grading system; HG, histological grade; NG, nuclear grade; CI, confidence interval; ALNs, axillary lymph nodes
