# Supplementary material for: Assessment of nuclear grade-based recurrence risk classification in patients with hormone receptor-positive, human epidermal growth factor receptor 2-negative, node-positive high-risk early breast cancer
Source: Breast Cancer. 2023 Aug 23;30(6):1054–64. doi: 10.1007/s12282-023-01500-2 (PMC10587205; doi:10.1007/s12282-023-01500-2)
Supplement: Supplementary file 2 — Supplementary file2 (DOCX 19 KB) [file 12282_2023_1500_MOESM2_ESM.docx]

Breast Cancer

**Assessment of nuclear grade-based recurrence risk classification in patients with hormone receptor-positive, human epidermal growth factor receptor 2-negative, node-positive high-risk early breast cancer**

Takeshi Murata^a*^, [tamurata@ncc.go.jp](mailto:tamurata@ncc.go.jp)

Masayuki Yoshida^b^ ,Sho Shiino^a^, Chikashi Watase^a^, Shohei Shikata^a^, Hiromi Hashiguchi^a^, Yukiko Yoshii^a^, Ayumi Ogawa^a^, Hirokazu Sugino^b^, Kenjiro Jimbo^a^, Akiko Maeshima^b^, Eriko Iwamoto^a^, Shin Takayama^a^, Akihiko Suto^a^,

^a^Department of Breast Surgery, National Cancer Center Hospital, 5-1-1 Tsukiji, Chuo-ku, Tokyo 104-0045, Japan.

^b^Department of Diagnostic Pathology, National Cancer Center Hospital, 5-1-1 Tsukiji, Chuo-ku, Tokyo 104-0045, Japan.

***Corresponding author**

Takeshi Murata

Department of Breast Surgery, National Cancer Center Hospital, 5-1-1 Tsukiji, Chuo-ku, Tokyo 104-0045, Japan

Tel: +81-3-3547-5201

Fax: +81-3-3542-3815

E-mail: [tamurata@ncc.go.jp](mailto:tamurata@ncc.go.jp)

ORCID: 0000-0003-0942-7599

**Online Resource Table 1. Recurrence events in patients who received NACT**

|  | All patients  (n=84) | Group 1  (n=72) | Group 2  (n=8) | Group 3  (n=2) | Group 4  (n=2) |
| --- | --- | --- | --- | --- | --- |
| IDFS events |  |  |  |  |  |
| Total IDFS events | 25 | 24 | 0 | 0 | 1 |
| Patients with invasive disease, first occurrence | 24 | 23 | 0 | 0 | 1 |
| Local/regional recurrence | 3 | 3 | 0 | 0 | 0 |
| Distant recurrence | 19 | 18 | 0 | 0 | 1 |
| Contralateral recurrence | 1 | 1 | 0 | 0 | 0 |
| Second primary neoplasm | 3 | 3 | 0 | 0 | 0 |
| All-cause mortality without invasive disease | 1 | 1 | 0 | 0 | 0 |
| DRFS events |  |  |  |  |  |
| Total DRFS events | 21 | 20 | 0 | 0 | 1 |
| Patients with distant relapse, any time | 19 | 18 | 0 | 0 | 1 |
| Bone | 6 | 6 | 0 | 0 | 0 |
| Liver | 10 | 9 | 0 | 0 | 1 |
| Lung | 4 | 4 | 0 | 0 | 0 |
| Brain | 2 | 2 | 0 | 0 | 0 |
| Lymph node | 8 | 8 | 0 | 0 | 0 |
| Pleura | 0 | 0 | 0 | 0 | 0 |
| CNS | 1 | 1 | 0 | 0 | 0 |
| Other^a^ | 0 | 0 | 0 | 0 | 0 |
| All-cause mortality without distant recurrence | 2 | 2 | 0 | 0 | 0 |

^a^Includes stomach (two), Median follow-up 71.4 months (IQR: 51.4–98.5)

Some patients were counted more than once in the subcategories if they had recurrences at different locations.

Group 1: Patients in cohort 1 by HG to cohort 1 by NG (i.e., no cohort conversion)

Group 2: Patients in cohort 2 by HG to cohort 2 by NG (i.e., no cohort conversion)

Group 3: Patients in cohort 3 by HG to cohort 3 by NG (i.e., no cohort conversion)

Group 4: Patients in cohort 2 or 3 by HG to cohort 1 by NG (i.e., cohort conversion).

Cohort 1: Patients with ≥4 positive ALNs or 1–3 positive ALNs and grade 3 or tumors ≥5 cm

Cohort 2: Patients with 1–3 positive ALNs, grade <3, tumor size <5 cm, and high Ki-67 index (≥20%)

Cohort 3: Patients with 1–3 positive ALNs, grade <3, tumor size <5 cm, and low Ki-67 index (<20%).

Abbreviations: IDFS, invasive disease-free survival; DRFS, distant relapse-free survival; CNS, central nervous system; IQR, interquartile range; NG, nuclear grade; HG, histological grade; NACT, neoadjuvant chemotherapy.
