# Supplementary material for: Assessment of nuclear grade-based recurrence risk classification in patients with hormone receptor-positive, human epidermal growth factor receptor 2-negative, node-positive high-risk early breast cancer
Source: Breast Cancer. 2023 Aug 23;30(6):1054–64. doi: 10.1007/s12282-023-01500-2 (PMC10587205; doi:10.1007/s12282-023-01500-2)
Supplement: Supplementary file 4 — Supplementary file4 (DOCX 21 KB) [file 12282_2023_1500_MOESM4_ESM.docx]

*Breast Cancer*

**Assessment of nuclear grade-based recurrence risk classification in patients with hormone receptor-positive, human epidermal growth factor receptor 2-negative, node-positive high-risk early breast cancer**

Takeshi Murata^a*^, [tamurata@ncc.go.jp](mailto:tamurata@ncc.go.jp)

Masayuki Yoshida^b^, Sho Shiino^a^, Chikashi Watase^a^, Shohei Shikata^a^, Hiromi Hashiguchi^a^,Yukiko Yoshii^a^,Ayumi Ogawa^a^, Hirokazu Sugino^b^, Kenjiro Jimbo^a^, Akiko Maeshima^b^, Eriko Iwamoto^a^, Shin Takayama^a^, Akihiko Suto^a^

^a^Department of Breast Surgery, National Cancer Center Hospital, 5-1-1 Tsukiji, Chuo-ku, Tokyo 104-0045, Japan.

^b^Department of Diagnostic Pathology, National Cancer Center Hospital, 5-1-1 Tsukiji, Chuo-ku, Tokyo 104-0045, Japan.

***Corresponding author**

Takeshi Murata

Department of Breast Surgery, National Cancer Center Hospital, 5-1-1 Tsukiji, Chuo-ku, Tokyo 104-0045, Japan

Tel: +81-3-3547-5201

Fax: +81-3-3542-3815

E-mail: [tamurata@ncc.go.jp](mailto:tamurata@ncc.go.jp)

ORCID: 0000-0003-0942-7599

**Online Resource Table 3. Clinicopahtological characteristics between patients with HG2-NG1/2 and patients with HG2-NG3**

|  | Category | HG2 – NG1/2  n=311 | HG2 – NG3  n=31 | *p*-value |
| --- | --- | --- | --- | --- |
| Tumor size, cm | <2 | 106 (34.1) | 12 (38.7) | 0.874 |
|  | 2–5 | 150 (48.2) | 14 (45.2) |  |
|  | ≥5 | 55 (17.7) | 5 (16.1) |  |
| Number of positive lymph nodes | 1–3 | 89 (28.6) | 10 (32.3) | 0.670 |
|  | ≥4 | 222 (71.4) | 21 (67.7) |  |
| Lymphovascular invasion | Negative | 130 (41.8) | 11 (35.5) | 0.496 |
|  | Positive | 181 (58.2) | 20 (64.5) |  |
| Ki-67 index, % | <20 | 167 (53.7) | 9 (29.0) | 0.009 |
|  | ≥20 | 114 (46.3) | 22 (71.0) |  |
| Ki-67 value | Median (IQR) | 19 (12.0–28.5) | 25 (18.9–34.6) | 0.002 |
| Tubule formation | Score 1 | 0 (0) | 5 (16.1) | <0.001 |
|  | Score 2 | 61 (19.6) | 26 (83.9) |  |
|  | Score 3 | 250 (80.4) | 0 (0) |  |
| Nuclear atypia | Score 1 | 3 (1.0) | 0 (0) | <0.001 |
|  | Score 2 | 265 (85.2) | 13 (41.9) |  |
|  | Score 3 | 43 (13.8) | 18 (58.1) |  |
| Mitotic counts | Score 1 | 191 (61.4) | 0 (0) | <0.001 |
|  | Score 2 | 120 (38.6) | 17 (54.8) |  |
|  | Score 3 | 0 (0) | 14 (45.2) |  |
| IDFS events | Total IDFS events | 52 (16.7) | 6 (19.4) |  |
|  | Patients with invasive disease, first occurrence | 36 (11.6) | 5 (16.1) |  |
|  | Local/regional recurrence | 11 | 1 |  |
|  | Distant recurrence | 25 | 4 |  |
|  | Contralateral recurrence | 3 | 0 |  |
|  | Second primary neoplasm | 14 | 1 |  |
|  | All-cause mortality without invasive disease | 2 | 0 |  |
| DRFS events | Total DRFS events | 32 (10.3) | 5 (16.1) |  |
|  | Patients with distant relapse, any time | 25 (8.0) | 4 (12.9) |  |
|  | Bone | 16 | 2 |  |
|  | Liver | 8 | 2 |  |
|  | Lung | 5 | 0 |  |
|  | Brain | 0 | 0 |  |
|  | Lymph node | 3 | 0 |  |
|  | Pleura | 1 | 0 |  |
|  | CNS | 0 | 0 |  |
|  | Other^a^ | 2 | 0 |  |
|  | All-cause mortality without distant recurrence | 7 | 1 |  |

^a^Includes stomach (two)

Abbreviations: IDFS, invasive disease-free survival; DRFS, distant relapse-free survival; NG, nuclear grade; HG, histological grade; CNS, central nervous system; IQR, interquartile range
